# Paracentrin 1, a synthetic antimicrobial peptide from the sea-urchin *Paracentrotus lividus,* interferes with staphylococcal and *Pseudomonas aeruginosa* biofilm formation

**DOI:** 10.1186/s13568-014-0078-z

**Published:** 2014-10-31

**Authors:** Domenico Schillaci, Maria Grazia Cusimano, Angelo Spinello, Giampaolo Barone, Debora Russo, Maria Vitale, Daniela Parrinello, Vincenzo Arizza

**Affiliations:** 1 Dip. STEBICEF, Università degli Studi di Palermo, Via Archirafi, Palermo, 32-90123, Italy; 2 IEMEST, Istituto Euromediterraneo di Scienza e Tecnologia, Via Emerico Amari, Palermo, 123-90139, Italy; 3 Istituto Zooprofilattico Sperimentale della Sicilia “A. Mirri”, Via G. Marinuzzi, Palermo, 3-90129, Italy

**Keywords:** AMP (Antimicrobial peptides), Biofilm, Staphylococci, Pseudomonas aeruginosa, Paracentrotus lividus

## Abstract

The rise of antibiotic-resistance as well as the reduction of investments by pharmaceutical companies in the development of new antibiotics have stimulated the investigation for alternative strategies to conventional antibiotics. Many antimicrobial peptides show a high specificity for prokaryotes and a low toxicity for eukaryotic cells and, due to their mode of action the development of resistance is considered unlikely. We recently characterized an antimicrobial peptide that was called Paracentrin 1 from the 5-kDa peptide fraction from the coelomocyte cytosol of the *Paracentrotus lividus*. In this study, the chemically synthesized Paracentrin 1, was tested for its antimicrobial and antibiofilm properties against reference strains of Gram positive and Gram negative. The Paracentrin 1 was active against planktonic form of staphylococcal strains (reference and isolates) and *Pseudomonas aeruginosa* ATCC 15442 at concentrations ranging from 12.5 to 6.2 mg/ml. The Paracentrin 1 was able to inhibit biofilm formation of staphylococcal and *Pseudomonas aeruginosa* strains at concentrations ranging from 3.1 to 0.75 mg/ml. We consider the tested peptide as a good starting molecule for novel synthetic derivatives with improved pharmaceutical potential.

## Introduction

Many natural antimicrobial peptides show a high specificity for prokaryotes and a low toxicity for eukaryotic cells, and for their mode of action the development of resistance by pathogenic bacteria is considered unlikely (Hancock and Rozek [2002]). At present, there has been an increase of interest in these molecules as potential new antimicrobials (Bax et al. [2000]; Mor [2000]).

In human medicine, chronic and persistent forms of some infectious diseases depend on the ability of pathogenic bacteria to develop bacterial communities called biofilms. Opportunistic pathogens, such as staphylococcal strains and *Pseudomonas aeruginosa* show a great ability to produce biofilms that preventing infected wounds to heal, render the treatment extremely challenging. In veterinary medicine, biofilms are believed to be involved in many diseases such as pneumonia, liver abscesses, enteritis, wound infections and mastitis, which is one of the most common diseases in dairy cattle (Clutterbuck et al. [2007])*. Staphylococcus aureus,* a major pathogen of mastitis has good in vitro antimicrobial susceptibility, but the therapy used to treat the affected animals is often disappointing and results in chronic infections due to the growth of bacteria as biofilms (Melchior et al. [2006]). The biofilm of *P. aeruginosa* is a severe complicating factor in bovine mastitis, which is often associated with contaminated udder washing water or contaminated intramammary dry-cow preparations (Melchior et al. [2009]).

The treatment of biofilm-associated infections is complicated because microbial biofilms are typically highly resistant to conventional antibiotics (Gilbert et al. [2002]). The discovery of anti-infective agents active not only against planktonic microorganisms but also against biofilms represents an important goal for an effective control of infections (Projan and Youngman [2002]).

The antimicrobial defence system of marine invertebrates is an interesting source of new anti-infective agents (Arizza [2013]). We focused, particularly, on the effector cells of the echinoderm immune system, the coelomocytes. In a recent study, the antimicrobial activity of a 5-kDa peptide fraction from coelomocyte cytosol (5-CC) of the *Paracentrotus lividus,* the sea-urchin from Mediterranean sea*,* was demonstrated in relation to a group of important human pathogens. The anti-biofilm activity of 5-CC was shown in *S. epidermidis* 1457, a clinical strain isolated from an infected central venous catheter, against reference staphylococcal biofilms and against *Candida albicans* and *Candida tropicalis* (Schillaci et al. [2010]; Schillaci et al. [2012]). We showed the presence of three principal peptides, in the 5-CC content, whose molecular weights were respectively 1251.7, 2088.1, and 2292.2. These peptides are the (9–19), (12–31), (24–41) fragments of a β-thymosin of *P. lividus*. We focused particularly on the smallest peptide, that we called paracentrin 1, 11 amino acids in length, because showed the common chemical-physical characteristics of an antimicrobial peptide (Wang and Wang [2004]).

The present study was aimed at evaluating the antibacterial and anti-biofilm activity of a chemically synthesized paracentrin 1 (SP1) against a group of staphylococcal reference strains and isolates and against *P. aeruginos*a ATCC 15442.

## Materials and methods

### Synthetic peptide

The SP1 was purchased from CASLO, Lyngby, Denmark utilizing the following sequence EVASFDKSKLK derived from ESI-MS analysis. The peptide was synthesized using Fmoc solid phase technology and the peptide content and purity was determined by high performance liquid chromatography (HPLC) and mass spectrometry (MS) analysis.

Net molecular charge was calculated by using the following formula (1) and the pI was calculated by using a web tool (http://isoelectric.ovh.org/).

(1) $$z=\sum_{i}N_{i}\frac{10^{pKa_{i}}}{10^{pH}}-\;\sum_{j}N_{j}\frac{10^{pH}}{10^{pH}+\;10^{pKa_{j}}}$$

Hydrophobic mean value was calculated by using the Liu-Deber hydrophobicity scale ([Liu and Deber 1998]). Secondary structure was evaluated utilizing the algorithm of Wang et al. ([2009]), present in antimicrobial peptide calculator web site (http://aps.unmc.edu/AP/prediction/prediction_main.php). The helical wheel projection was performed using the Gautier et al. ([2008]) algorithm present in the HeliQuest site (http://heliquest.ipmc.cnrs.fr) with a window size of 11 residues.

### Microbial strains

The staphylococcal reference strains used were: *Staphylococcus aureus* ATCC 29213, *Staphylococcus aureus* ATCC 25923, *Staphylococcus aureus* ATCC 6538, and *Staphylococcus epidermidis* RP62A, known for its ability to form a biofilm (Schumacher-Perdreau et al. [1994]). Four staphylococcal isolates from the Istituto Zooprofilattico Sperimentale, Sicily (IZS) bacterial collection including strain 657 isolated from a milk sample from an individual sheep affected by mastitis, strain 688 and 700 and strain 702 isolated from bulk milk samples from different sheep flocks. The isolates were selected on blood agar plates and on Mannitol Salt Agar (Difco, Sparks, MD). The colonies were typed by API Staph strip (bio-Mérieux) and tube coagulase test was performed (Canicatti and Roch [1989]).

*Pseudomonas aeruginosa* ATCC 15442, the reference strain in official tests for antibacterial evaluation in vitro (UNI EN European Standard), was also used in this study.

### Minimum inhibitory concentrations (MIC)

MICs were determined by a micro-method described previously (Schillaci et al. [2005]). Briefly, a series of solutions of SP1 was prepared with a range of concentrations from 25 to 0.07 mg/ml(obtained by two-fold serial dilution). The serial dilutions were made in Tryptic Soy Broth (TSB) (Merck) in a 96-wells plate; to each well was added 10 μl of a bacterial suspension from a 24 h culture containing ~1 × 10^6^ CFU/ml. The plate was incubated at 37°C for 24 h; after this time, the MICs were determined by a microplate reader (ELX 800, Bio-Tek Instruments), and defined as the lowest concentration of compound whose O.D., read at 570 nm, was comparable with the negative control wells (broth only, without inoculum).

### Evaluation of Biofilm formation

All the bacterial reference strains were tested for their ability to form biofilms. Briefly, bacteria were grown in Tryptic Soy Broth (TSB, Sigma) containing 2% glucose overnight at 37°C in a shaking bath and then diluted 1:200 to a suspension with optical density (OD) of about 0.040 at 570 nm corresponding to ~10^6^ CFU/ml. Polystyrene 24-well tissue culture plates were filled with 1 ml of diluted suspension and incubated for 24-hours at 37°C. Then, the wells were washed three times with 1 ml of sterile phosphate-buffered saline (PBS) and stained with 1 ml of safranin 0.1% v/v for 1 min. The excess stain was removed by placing the plates under running tap water. Plates were dried overnight in inverted position at 37°C. Safranin stained adherent bacteria in each well were re-dissolved to homogeneity in 1 ml of 30% v/v glacial acetic acid, and the OD was read at 492 nm. Each assay was performed in triplicate and repeated at least twice.

### Biofilm prevention assay

Procedure described above was used to evaluate the activity of SP1 in preventing biofilm formation. Polystyrene 24-well tissue culture plates were filled with 1 ml of diluted bacterial suspension (OD of about 0.040 at 570 nm), obtained and diluted as previously seen, and sub-MIC concentrations, ranging from 6.2 to 0.7 mg/ml, of SP1 were directly added to the bacterial suspension at time zero and incubated at 37°C for 24 hours. After that time the wells were washed and stained with safranin as seen in biofilm forming assay. By comparing the average optical density of the growth in control wells with that in the sample wells, the following formula was used to calculate the percentages of inhibition for each concentration of the sample:

(2) $$\mathrm{Inhibition}\;\left(\%\right)=\frac{{OD}_{570}\;\mathrm{growth}\;\mathrm{control}-{OD}_{570}\;\mathrm{sample}}{{OD}_{570}\;\mathrm{growth}\;\mathrm{control}}\times 100$$

Each assay was performed in triplicate and assays were repeated at least twice.

### Scanning electron microscopy

The effects of SP1 on formation of bacterial biofilm were morphologically evaluated by scanning electron microscopy (SEM). Glass slides in the bottom of a polystyrene 24-well tissue culture plates, were filled with 1 ml of a *S. epidermidis* RP62A suspension, obtained and diluted as previously seen, and a sub-MIC concentration of 3.1 mg/ml of SP1 was directly added at time zero and incubated at 37°C for 24 hours. After that time, the glass slides were gently washed four times with PBS to remove non-adherent bacteria and fixed with 2.5% glutaraldehyde-2% paraformaldehyde in 0.1 M cacodilate buffer (pH 7.4) for 30 min at 4°C. The bacterial preparation were washed with phosphate saline buffer PBS and post-fixed in osmium tetroxide 1% for 30 min at 4°C, followed by an ethanol dehydration series: 15 minutes in 50:50 ethanol: H_2_O, 15 minutes in 75:25 ethanol: H_2_O, 15 minutes in 95:5 ethanol: H_2_O, and 30 minutes in 100% ethanol than a critical point drying procedure was followed, and the preparations were mounted on aluminium stubs, and gold coated in a sputter coater. Imaging was conducted with a LEO 420 scanning electron microscope as previously reported (Arizza et al. [2011]).

### Haemolytic assay

The assay was performed as described (Arizza et al. [2013]). In brief, freshly collected rabbit erythrocytes (RE) with heparin, kindly provided by the “Zooprophylaxis Institute of Sicily” (Palermo, Italy), were washed (400 g for 10 min at 4°C) to remove the buffy coat, and the erythrocytes obtained were washed three times with phosphate-buffered saline (PBS: 6 mM KH_2_PO_4_; 30 mM Na_2_HPO_4_; 0.11 M NaCl; pH 7.4) and suspended in 10 ml PBS to obtain an 80 × 10^6^ cells/ml suspension. Aliquots of 200 μL of RE suspension were mixed with 200 μl of SP1 concentrations (1.5, 3.2, 6.2 and 50 mg/ml) prepared in PBS, two-fold sequentially diluted v/v with a PBS. After an 1 h incubation at 37°C, the reaction mixture was centrifuged at 800 g for 15 min at 4°C to remove debris and residual erythrocytes. The O.D. of haemoglobin release was measured spectrophotometrically at a 541 nm wavelength. Spontaneous haemoglobin release (0%) was estimated incubating the RE with PBS while the complete haemolysis (100%) was assessed incubating the erythrocytes in a solution of 0.1% Triton-X 100 in distilled water and the haemolysis percentage was calculated according to the following equation:

(3) $$\mathrm{Hemolysis}\;\left(\%\right)=\frac{{OD}_{541}Hb\;\mathrm{release}\;\mathrm{in}\;\mathrm{the}\;\mathrm{reaction}\;\mathrm{mixture}-{OD}_{541}\mathrm{spontaneous}\;Hb\;\mathrm{release}\;}{{OD}_{541}\;\mathrm{complete}\;Hb\;\mathrm{release}}\times 100$$

### Molecular dynamics simulations

The molecular folding of the peptide in aqueous solution was investigated *in silico* by molecular dynamics (MD) simulations, following recently reported procedures (Lauria et al. [2014]; Lentini et al. [2014]). In details, a 400 ns of MD simulation was carried out at 300 K, in the explicit water solvent and in the presence of 150 mM Na^+^ and Cl^−^ counterions, using the Amber99SB-ILDN force field (Lindorff-Larsen et al. [2010]) implemented in the GROMACS 4.6.5 software package (Pronk et al. [2013]).

## Results

### Molecular Dynamics of SP1

SP1 is an 11-residue-long cationic peptide mainly enriched by residues such as lysine, with a pI of 10.72 and a net charge of +1 at pH 7.0.

**Figure 1:**
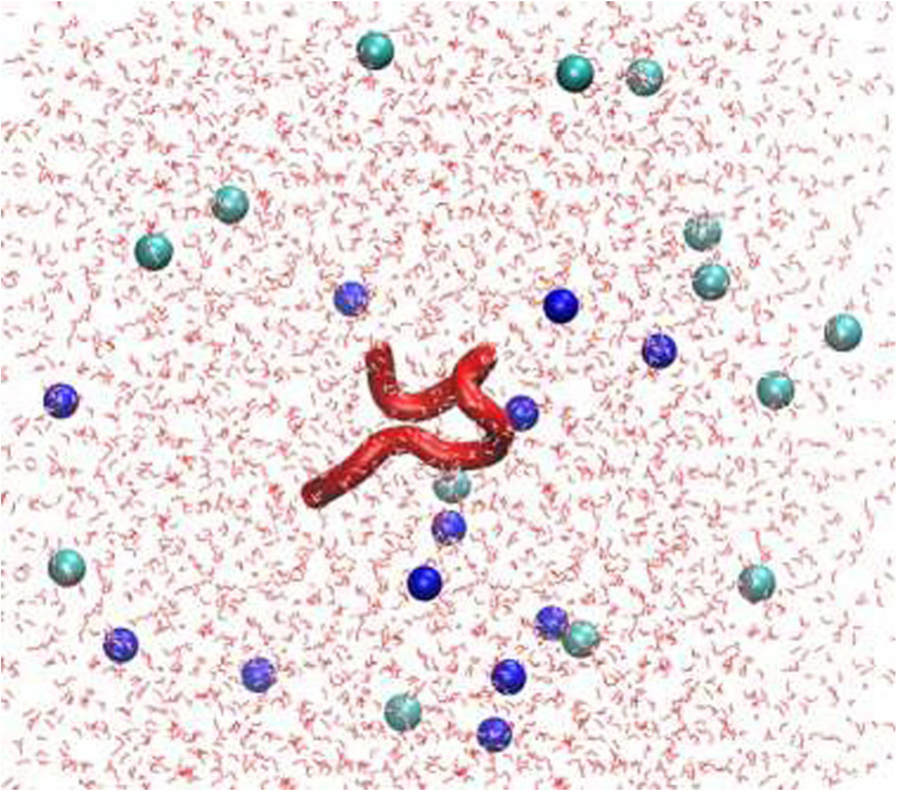
**Example of MD simulation box, showing the SP1 molecule, the solution counterions, Na**^**+**^**(in blue) and Cl**^**−**^**(in cyan), and the explicit water molecules (in red).**

**Figure 2:**
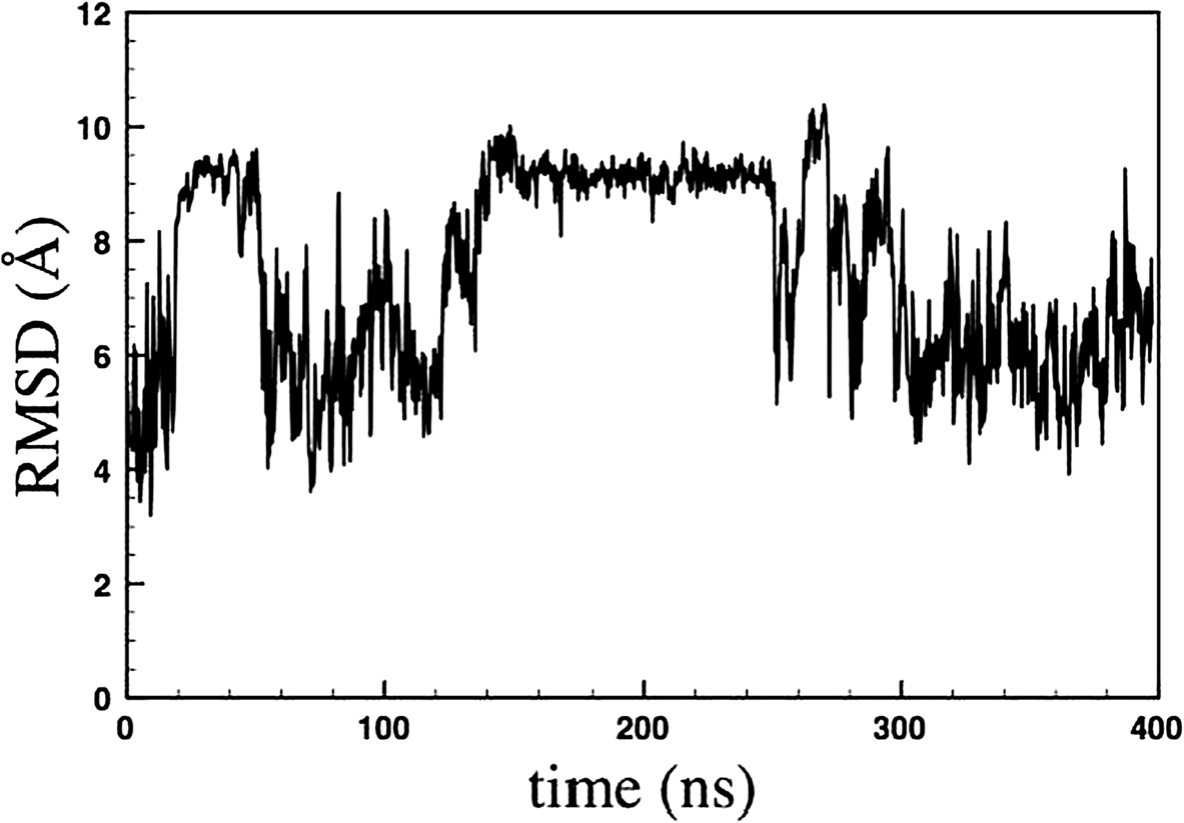
Plot of the RMSD obtained for SP1 up to 400 ns of MD simulation.

**Figure 3:**
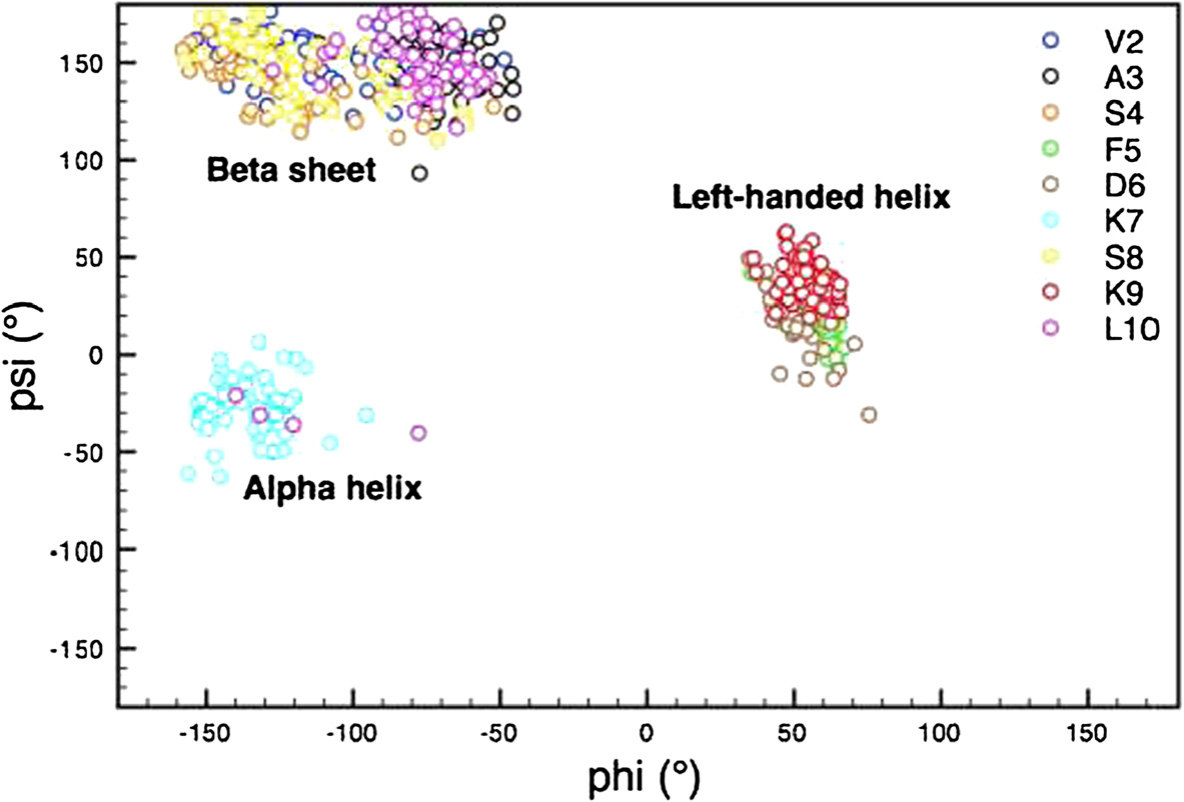
Ramachandran plot showing the values of psi and phi angles assumed by residues 2–10 of SP1 between 150 and 250 ns of the MD simulation.

**Figure 4:**
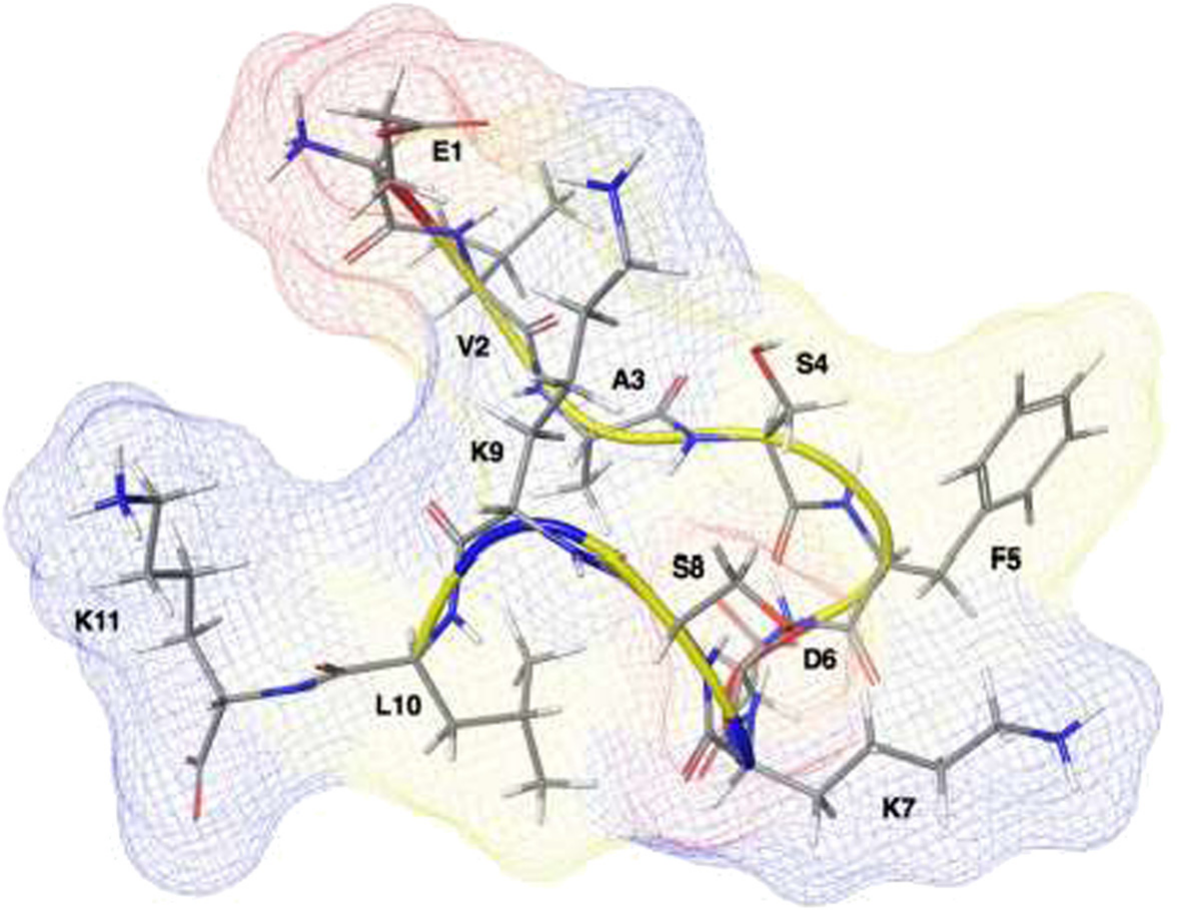
**Molecular representation of SP1 at about 200 ns of MD showing the non amphipathic structure of the peptide.** The potential surface is superimposed. Color code: acidic residues in red, basic residues in blue, and hydrophobic residues in yellow.

To investigate SP1 in physiological conditions, we performed a MD simulation using the model represented in Figure 1. The *in silico* study provided a comprehensive picture of the dynamic equilibrium existing among the possible backbone conformations of the small oligopeptide. In fact, the root mean square deviations (RMSD) in Figure 2 show that the peptide conformation continuously and consistently changes during the 400 ns of simulation. Essentially all possible backbone conformations are assumed. However, interestingly, between 150 and 250 ns the structure is more stable and preserved, before becoming again flexible and random. To analyse this time region quantitatively, we have reported the Ramachandran plot in Figure 3 of snapshots sampled every 10 ns. This plot shows that in this time range the different residues assume preferential local conformations. In particular, residues 2, 3, 4 and 8 are in the beta sheet conformation, residues 5, 6 and 9 are in the left-handed helix conformation, residue 7 is in the alpha helix conformation, while residue 10 is in the beta sheet conformation and only sporadically in the alpha helix conformation. The corresponding structure is depicted in Figure 4. The results obtained allow us to confirm that polar and apolar residues are not segregated on opposing surfaces through the long axis of the oligopeptide. In this peculiar conformation, the one that most frequently occurs in the investigated simulation time, the peptide possesses a hydrophobic non-amphipathic core constituted by V^2^, A^3^, S^4^, F^5^ with a hydrophobic region placed between E^1^ and D^6^. These features confer to SP1 properties similar to the AMPs with hydrophobic core.

### Antibacterial activity of SP1

SP1 was tested at concentrations ranging from 25 to 0.07 mg/ml against a group of Gram positive and Gram negative reference strains. The antibacterial activity of SP1, expressed as minimum inhibitory concentrations (MICs) against planktonic cells of staphylococcal reference strains and isolates from veterinary origin and *P. aeruginosa* is listed in Table 1. All bacterial strains showed susceptibility to the antibacterial action of SP1. In particular the strain of *S. epidermidis* RP62A resulted to be the most susceptible, indeed, the MIC value was 6.2 mg/ml, while others attested to a value of 12.5 mg/ml. SP1 was active against all isolates of animal origin strains and in particular showed the highest activity against the strain #657 with a value of 6.2 mg/ml (Table 1).

**Table 1:** MIC values of SP1 tested against bacterial strains

**Bacterial strains**	**MIC (mg/ml)**
*S. aureus* ATCC 25923	12.5
*S. aureus* ATCC 29213	12.5
*S. aureus* ATCC 6538	12.5
*S. epidermidis* RP62A	6.2
*S. aureus* 100	12.5
*S. aureus* 657	6.2
*S. aureus* 700	12.5
*S. aureus* 702	12.5
*P. aeruginosa* ATCC15442	12.5

MIC values for each strain are the means of three different assays performed in duplicate

### Interference with biofilm formation

**Figure 5:**
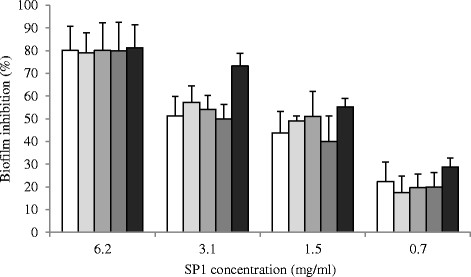
**Interference with biofilm formation of SP1 against reference staphylococcal and*P. aeruginosa*strains.** The percentage of inhibition were evaluated comparing the samples with not-treated 24 h old biofilms and staining with safranin. Bacterial strains () *S. aureus* 25923, () *S. aureus* 29213, () *S. aureus* 6538, () *S. epidermidis* RP62A, () *P. aeruginosa* 15442. Data for each strain are the mean of three distinct experiments ± S.D.

The interference with biofilm formation of SP1 against staphylococcal reference strains as *S. aureus* 25923, *S. aureus* 29213, *S. aureus* 6538, *S. epidermidis* RP62A and *P. aeruginosa* 15442 was observed. The inhibition was very evident to the highest concentrations of SP1 at 6.2 mg/ml, when the values, for all strains, reached approximately 80%. At the lowest concentrations of SP1 the degree of inhibition is reduced by following a dose dependence. *P. aeruginosa* strain 15442 was the most sensitive inhibitory activity of SP1, in fact, at a concentration of 3.1 mg/ml, a value of about 73% inhibition was observed, while for the other strains the inhibition values were of around 50% (Figure 5).

**Figure 6:**
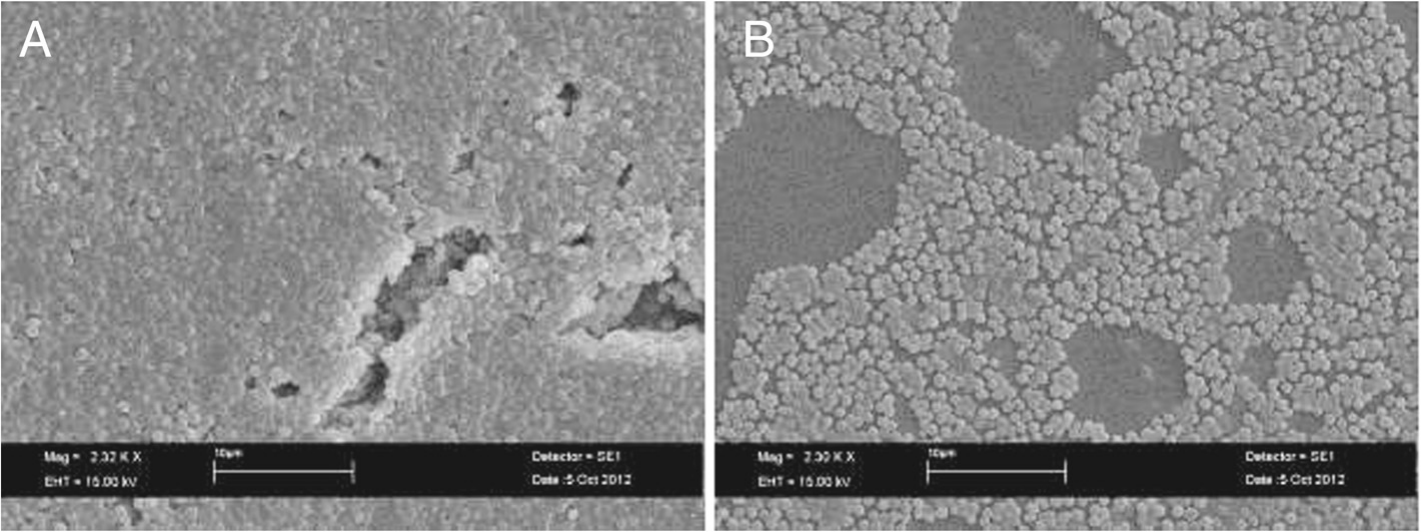
**SEM micrography showing the effect of sub-MIC concentration of SP1 on*S. epidermidis*RP62A biofilm formation. A)** Growth control (not treated with SP1); **B)** sample treated with a sub-MIC concentration (3.1 mg/ml) of SP1.

SEM analysis was carried out to visualize the detailed architecture of biofilm and the antibiofilm effects of SP1 on bacterial biofilm of *S. epidermidis* RP62A (Figure 6). The control biofilm after 24 h was composed by multilayered conglomerated bacterial cells clusters that produced a dense biofilm matrix (Figure 6A). Sub MIC concentration (3.1 mg/ml) of SP1 was able to inhibit the formation of a multistratified structure, in fact in the sample treated with SP1 we found few monostratified bacterial cell clusters respect to the growth control (Figure 6B).

### Haemolytic assay

**Figure 7:**
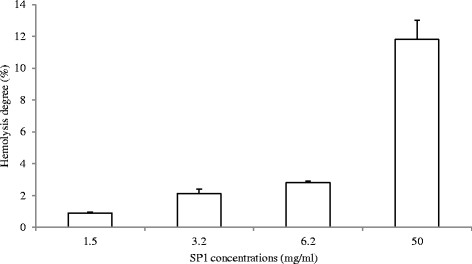
**Hemolytic activity of SP1 peptides from*Paracentrotus lividus*hemocytes against rabbit blood cells at different concentrations: 1.5 mg/ml; 3.2 mg/ml; 6.2 mg/ml; 50 mg/ml.** Data are the mean value of three separate experiments and expressed as percentage of hemolysis ± SD

The haemolytic activity of antimicrobial peptides against mammalian erythrocytes is often used as a preliminary evaluation of their selective toxicity and of the interactions of the cationic peptides with negatively charged membranes (Fischer et al. [2003]; Wu et al. [2010]) The performed hemolytic experiment to evaluate the interaction and the potential toxicity of the peptides SP1 did not show a measurable toxic effect against RBC (~1%) at MIC concentrations. A slight haemolytic activity, about 11.8%, was evident only at the highest concentration (50 mg/ml) (Figure 7).

## Discussion

The echinoderms are considered a good source for AMPs and a variety of peptides with antimicrobial properties have been isolated from them. Antimicrobial activity has been reported in gonads of the asteroid *Marthasterias glacialis* (Stabili and Pagliara [1994]), and *Paracentrotus lividus* contains, in the low molecular weight fraction (<5 kDa) of acid precipitate of their coelomocytes, peptides with antimicrobial activity against staphylococcal biofilms. In addition, we recently reported that immune mediators cells in the echinoderm *Holothuria tubulosa* is a source of novel AMPs with anti-staphylococcal biofim activity (Schillaci et al. [2013]). Moreover, two cystein-rich AMPs, named centrocyns, have been characterized in the green sea-urchin *Strongylocentrotus droebachiensis* (Li et al. [2010]).

The synthetic fragment SP1 of β-thymosin extracted from coelomocytes of the *P. lividus* shares structural characteristics of many antimicrobial peptides: it is a cationic peptide and possesses a significant proportion (~40%) of hydrophobic or non polar residues (Hancock and Lehrer [1998]; Zasloff [2002]). Although the α-helix is the most common secondary structure for the antimicrobial activity of AMPs (Mor and Nicolas [1994]; Skerlavaj et al. [1996]; Storici et al. [1994]; Tossi et al. [1994]) the *in silico* study suggests that the conformation assumed by SP1 (Figure 4) might be responsible for the observed antimicrobial activity. The helix structure of echinoderm AMPs shares a characteristic amphipathic structure with alternating hydrophobic and polar residues along the primary structure. According to the Shai-Matsuzaki-Huang model, the peptides bind to the membrane surface first and then with their amphipathic structure they enter into the membrane, breaking up the lipid chains and forming transient pore; this process can cause a collapse of the membrane at a critical peptide concentration (Hancock and Chapple [1999]; Huang [2000]; Matsuzaki [1998]; Shai [2002]). The SP1 peptide has a structure very different from other echinoderm AMPs (Schillaci et al. [2013]; Schillaci et al. [2012]). Moreover, such structure, unexpectedly, does not have an amphipathic nature with a hydrophobic face opposite to a hydrophilic one, because the polar charged residues and those hydrophobic are not arranged uniformly. The peptide contains a sequence of four hydrophobic/non polar residues which contribute to constitute a hydrophobic core, flanked at both ends by cationic and polar residues that can solubilize the peptides in aqueous solution providing a binding site for bacterial membranes. This particular conformation is different from other antimicrobial peptides, designed as transmembrane mimetic models and that spontaneously become inserted into the cell membranes (Chan et al. [2004]; [Liu and Deber 1998]; Stark et al. [2002]).

We found that the SP1 showed a broad antimicrobial activity against important pathogens such as *S. aureus* and *P. aeruginosa* but it acts at high concentration (12.5 or 6.2 mg/ml) against planktonic forms of these two microorganisms. Such weak activity is comparable to that reported for a different Echinoderm, *Holothuria tubulosa* (Schillaci et al. [2013]) and to that of some described innate human defence protein like lactoferrin in vitro (de Andrade et al. [2014]). MD results suggest that the observed weak activity could be due to the low stability of SP1, in fact, as showed in RMSD plot (Figure 2), the molecule is stable only in a limited time period and in this period it can present the active conformation of Figure 1. However, SP1 shows an interesting additional antimicrobial effect interfering with biofilm formation *in vitro* of the above cited pathogens*,* at lower concentrations than MIC evaluated against the planktonic forms. The prevention of biofilm formation – rather than its elimination – is the best strategy to contrast the growth, as a sessile community, of many pathogens.

We do not know the antimicrobial mechanisms of SP1, but we could speculate as for other cationic non-amphipathic microbial peptides that the positive charges carried by the peptide are essential for the membrane binding through electrostatic interaction between residues with anionic phospholipids. The peptide-membrane interaction could be responsible for membrane aggregation by peptides bridging simultaneously two membranes or for negative curvature of the membrane asymmetry that can form tubes (Lamaziere et al. [2007]). The synthetic SP1 could act like Dermaseptin S9, a non-amphipathic antimicrobial peptides produced by the skin of the South American hylid frog, *Phyllomedusa sauvagei*, that contains, centrally located, a hydrophobic core that can insert the peptide in interior membrane (Lequin et al. [2006]). Dermaseptin S9 exerts a microbicidal activity by perturbating both the membrane interface and the hydrophobic core of the bacterial membrane (Auvynet et al. [2008]).

In a preliminary evaluation of selective toxicity, it was gratifying to note the general lack of hemolysis of rabbit erythrocytes by SP1, even at peptide concentrations up to 50 mg/ml. Such selectivity of cationic non amphipathic antimicrobial peptides for bacterial membranes may be explicable, in part, by the differences in the compositions of eukaryotic and prokaryotic membranes (Matsuzaki [1999]; Zasloff [2002]). The outer leaflet of mammalian cells is predominantly composed of the zwitterionic phospholipids phosphatidylcholine and sphingomyelin (Verkleij et al. [1973]), along with cholesterol (Turner and Rouser [1970]), while bacterial membranes contain mainly anionic phospholipids and no cholesterol (Brock [1974]). In addition, the outer surfaces of Gram-negative bacteria contain lipopolysaccharides, while those of Gram-positive bacteria contain teichoic acid, which in both cases add to the negative charge of the bacterial surface (Brock [1974]). The cationic nature of native antimicrobial peptides clearly contributes to their preferential recognition by the negatively charged outer surfaces of bacterial membranes (Oren and Shai [1998]; Shai [1999]).

The extensive clinical use of classical antibiotics has led to the growing emergence of many medically relevant resistant strains of pathogens (Patel [2005]). Therefore, the development of a new class of antimicrobials with a different mechanism of action than conventional antibiotics has become critical. The cationic antimicrobial peptides could represent such a new class (Andreu and Rivas [1998]; Hancock [1997]; Sitaram and Nagaraj [2002]). The development of resistance to membrane active peptides whose sole target is the cytoplasmic membrane is not expected because this would require substantial changes in the lipid composition of cell membranes of microorganisms (Hancock [2001]).

The anti-adhesion property showed by SP1 against *S. aureus* and *P. aeruginosa* strains is very interesting considering that the two opportunistic pathogens are able to form biofilms in open wounds, such as chronic diabetic foot ulcers, or infected wounds in clinical and veterinary medicine. New antimicrobial agents that are effective against staphylococci and *P. aeruginosa* to treat infected wounds are needed, and a potential topical application of SP1 could be supposed. Moreover, the two species are also involved in food spoilage and biofilm formation of food transmitted pathogens and an application of SP1 in the food processing is possible and closer, at this stage of our study, than application in clinical or veterinary health.

A chemotherapeutic approach combining conventional antibiotics and novel anti-biofilm agents could be a new strategy for the treatment of biofilm-associated infections like mastitis in veterinary field or the topical treatment of infected wounds in clinical and veterinary setting.

Finally, the tested synthetic peptide is a good starting point to design new synthetic derivatives with modified chemical-physical properties, with the aim to improve their antimicrobial activity against pathogens and their pharmaceutical potential (Brogden and Brogden [2011]; Huang et al. [2010]).

## Competing interests

The authors declare that they have no competing interests.
